# From host individual traits to community structure and composition: *Bartonella* infection insights

**DOI:** 10.1186/s13071-024-06523-y

**Published:** 2024-10-28

**Authors:** Gabriella Lima Tabet Cruz, Jonathan Gonçalves-Oliveira, Elba Regina Sampaio de Lemos, Paulo Sergio D’Andrea, Cecilia Siliansky de Andreazzi

**Affiliations:** 1 Laboratório de Biologia e Parasitologia de Mamíferos Silvestres Reservatórios (LABPMR), Instituto Oswaldo Cruz, Fundação Oswaldo Cruz (Fiocruz), Rio de Janeiro, Brazil; 2 Pró-Reitoria de Pós-Graduação, Pesquisa e Inovação (PROPGPI), Universidade Federal do Estado do Rio de Janeiro (Unirio), Rio de Janeiro, Brazil; 3 Laboratório de Hantaviroses e Rickettsioses, Instituto Oswaldo Cruz, Fundação Oswaldo Cruz (Fiocruz), Rio de Janeiro, Brazil; 4 Laboratory for Zoonotic and Vector-Borne Diseases, Koret School of Veterinary Medicine, The Hebrew University of Jerusalem, Rehovot, Israel; 5 International Platform for Science, Technology and Innovation in Health, PICTIS, Fiocruz, Ílhavo, Portugal; 6 Departamento de Biodiversidad, Ecología y Evolución, Universidad Complutense de Madrid, Madrid, Spain

**Keywords:** *Bartonella*, Functional diversity, Host trait, Interaction network, Mammal, Phylogeny

## Abstract

**Background:**

Phylogeny, combined with trait-based measures, offers insights into parasite sharing among hosts. However, the specific traits that mediate transmission and the aspects of host community diversity that most effectively explain parasite infection rates remain unclear, even for the *Bartonella* genus, a vector-borne bacteria that causes persistent blood infections in vertebrates.

**Methods:**

This study investigated the association between rodent host traits and *Bartonella* infection, as well as how rodent community diversity affects the odds of infection in the Atlantic Forest, using generalized linear models. Additionally, we assessed how host traits and phylogenetic similarities influence *Bartonella* infection among mammal species in Brazil. To this end, rodents were sampled from ten municipalities in Rio de Janeiro, southeastern Brazil. Then, we calculated several diversity indices for each community, including Rényi’s diversity profiles, Fisher’s alpha, Rao’s quadratic entropy (RaoQ), Functional Diversity (FDis), Functional Richness (FRic), and Functional Evenness (FEve). Finally, we compiled a network encompassing all known interactions between mammal species and *Bartonella* lineages recorded in Brazil.

**Results:**

We found no significant relationship between diversity indices and the odds of *Bartonella* infection in rodent communities. Furthermore, there was no statistical support for the influence of individual-level traits (e.g., body length, sex, and age) or species-level ecological traits (e.g., locomotor habitat, dietary guild, and activity period) on *Bartonella* infection in rodents. A country-scale analysis, considering all mammal species, revealed no effect of host traits or phylogeny on *Bartonella* infection.

**Conclusions:**

This study highlighted wild mammals that share *Bartonella* lineages with livestock, synanthropic, and domestic animals, underscoring the complexity of their maintenance cycle within the One Health framework. A key question arising from our findings is whether molecular host–cell interactions outweigh host body mass and ecological traits in influencing *Bartonella* infection, potentially opening new avenues for understanding host–parasite relationships and infection ecology.

**Graphical Abstract:**

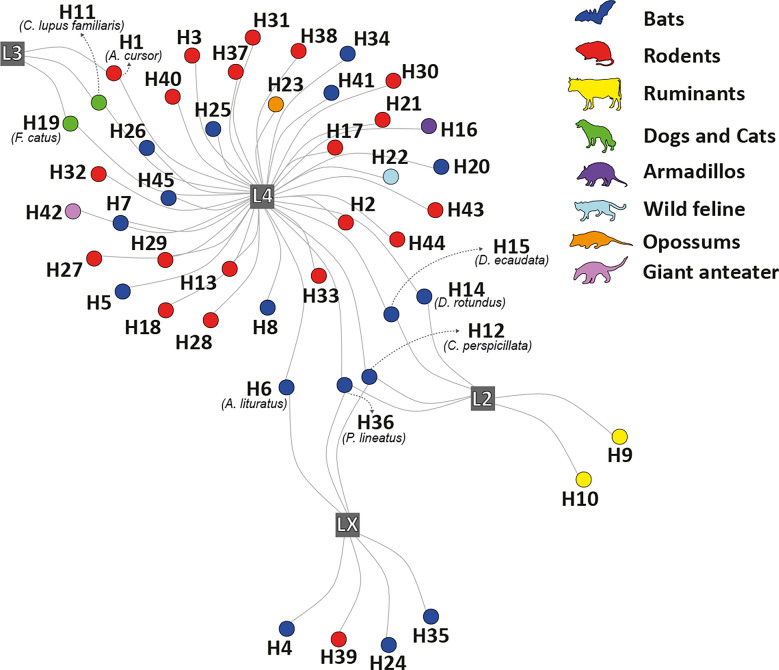

**Supplementary Information:**

The online version contains supplementary material available at 10.1186/s13071-024-06523-y.

## Background

Host evolutionary history, combined with trait-based measures, is associated with parasite spread among hosts [1, 2]. Variations in host traits modulate vector exposure, parasite encounters, and pathogen spread within local host communities [3–5]. Therefore, functional diversity measures, which encompass the variety and distribution of ecological, morphological, and physiological traits within a community, can serve as an adequate proxy for the structural role of host communities [6, 7]. However, it remains unclear whether and which functional diversity indices can serve as an indicator of infection rates [5, 6, 8–10].

The *Bartonella* genus consists of facultative intracellular alphaproteobacteria and vector-borne pathogens that can cause persistent hemotropic infections in their vertebrate hosts [11]. From an ecological perspective, *Bartonella* infects a broad diversity of host species, with varying levels of specificity across host phylogeny. Even host-specific species, such as *B. henselae*, which are commonly found in domestic cats, have also been identified in dogs and wild mammals in Brazil [12, 13]. Furthermore, these bacteria have been detected in various mammalian orders and ectoparasites worldwide, including Argentina [14], Chile [15], Colombia [16], Israel [17], the USA [18], Italy [19], Thailand [20], Japan, Russia, Korea, and Taiwan [21].

*Bartonella* spp. exhibit a phylogenetic pattern that separates them into lineages, each displaying a distribution of virulence factors that contribute to their persistence and pathogenicity [22]. These virulence factors show evolutionary patterns in host specificity, with certain lineages closely associated with specific mammal orders, such as lineage 2 with ruminants and lineage 4 with rodents [22–24]. Thus, these bacteria provide a suitable system for studying disease–diversity relationships.

Rodent species are frequently reported as hosts for many zoonotic agents [25] and also harbor a high diversity of *Bartonella* species [15, 26, 27]. Certain traits of rodent species, such as age at sexual maturity, short gestation periods, and large litter sizes, traits associated with fast life history strategies, can influence the risk of infection by zoonotic agents [25, 28]. Therefore, a trait profile approach to known *Bartonella* host species may allow us to forecast which species are likely to act as reservoirs for these bacteria.

In this context, the aim of this study was threefold. First, data on *Bartonella* infection in rodent communities were used to (i) investigate which host traits at the individual level are most associated with the odds of *Bartonella* infection and (ii) examine how community diversity affects the odds of infection in Atlantic Forest areas. Second, for host species of different mammalian orders that occur in Brazil, (iii) our objective was to assess whether the probability of *Bartonella* infection is influenced by traits and phylogenetic similarities between these host species.

In the individual and species-level analyses, we hypothesized that infection would be more frequent among hosts with similar traits. Morphological traits such as body length, body mass, and tail length are related to individual age and lifetime exposure to parasites [29, 30]; therefore, we expected these individual measures to be positively related to infection probability. Furthermore, since male rodents tend to increase their mobility during reproductive periods and may experience hormone-induced immunosuppression [31], we anticipated that males would have a higher probability of *Bartonella* infection compared with females. We also hypothesized that scansorial or semi-scansorial locomotor habitats and an invertebrate diet would increase the probability of infection, as these traits increase the likelihood of encountering a vector [32, 33]. Finally, because activity period is associated with resource sharing and is evolutionarily related to other ecological traits such as foraging strata [34], we expected cathemeral rodents, which are active both day and night, to face a higher risk of exposure due to overlap activity periods with vectors and other hosts.

In the community analysis, we expected that at greater functional divergence [i.e., Functional Evenness index (FEve)], which may be associated with a reduced abundance of highly competent species, would result in lower odds of *Bartonella* infection. Conversely, higher functional diversity [that is, Functional Diversity (FDis), Functional Richness (FRic), and Rao’s quadraticentropy (RaoQ) indices] was expected to increase the chance of *Bartonella* infection by enhancing trait diversity and the abundance of potential host species. Definitions of these functional diversity indices are provided in the Methods subsection titled Host community structure.

Finally, considering that *Bartonella* lineages have functional factors related to pathogenicity [22], we also aimed to examine whether host traits at the species level influence the sharing of *Bartonella* lineages among hosts of different mammalian orders. By integrating analyses across these scales, we can achieve a comprehensive understanding of how individual- or species-level traits, community diversity, and phylogenetic relationships affect parasite infection [6, 7]. This integrated approach facilitates the development of more effective monitoring, management, and control strategies tailored to specific ecological contexts and host community structures [35].

## Methods

Two distinct datasets were used to investigate our hypotheses on three scales: individual, community, and species. For the first set of analyses, we used data from several rodent communities sampled across the Brazilian Atlantic Forest, including individual-level information on life history traits and *Bartonella* infection. This biome was chosen due to the higher prevalence of these bacteria in rodents compared with other Brazilian biomes [36]. For the second set of analyses, we expanded the scale to include all other mammalian orders occurring in Brazil. Consequently, we analyzed traits at the species level and *Bartonella* infection, as there is no individual-level database available.

### Study area and rodent community data source

**Figure 1:**
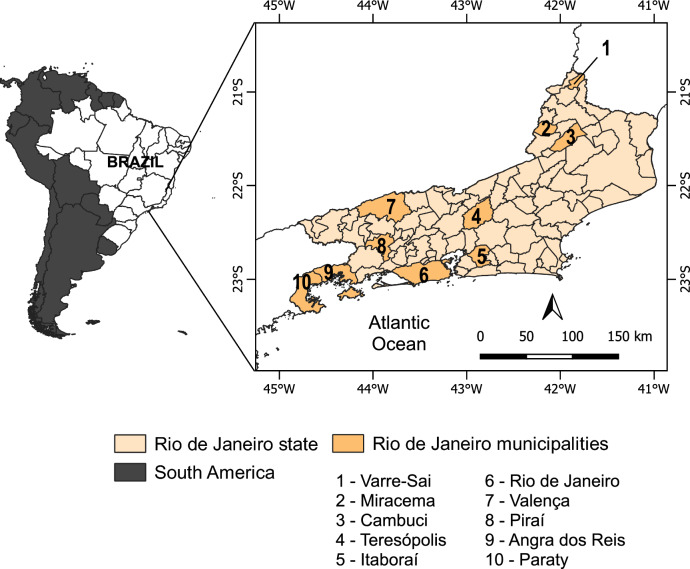
Study areas for wild rodents in the state of Rio de Janeiro, Brazil (2004–2019)

We used geo-located data from previous studies on individual rodents, infected or not with *Bartonella*, in ten municipalities in the state of Rio de Janeiro, southeastern Brazil (see Fig. 1) [37, 38]. Additional data were gathered from a research project on the biodiversity of Atlantic Forest, covering rodent captures from 2004 to 2019 in the same areas. A unified dataset was compiled, containing information on 398 rodent individuals. Rodent species were identified by morphological examination, karyotyping, and molecular analyses by experts from the Laboratory of Biodiversity and Parasitology of Wild Mammal Reservoirs, following the methodology outlined by D’Andrea et al. [39]. The number of individuals sampled in each community ranged from 12 to 138, with 3–8 rodent species. All procedures involving rodents were approved in advance by the Institutional Ethics Committee of Animal Research at the Oswaldo Cruz Foundation under the license number LW39/14.

*Bartonella* was detected using polymerase chain reaction (PCR) methods and DNA sequencing. Bacterial DNA was extracted from liver and spleen samples and screened by PCR targeting the *gltA*, *rpoB*, *ftsZ*, and *groEL* genes. In total, 32 rodent individuals with the *Bartonella* gene were detected, with the number of infected individuals in each community ranging from 0 to 8. Detailed information on the study area, sampling methods, and molecular assays can be found in Rozental et al. [37] and Gonçalves-Oliveira et al. [38].

### Host trait data

Data on morphological and ecological traits associated with host life history were obtained from our database, the EltonTraits database [40], and from Paglia et al. [41]. The morphological data from our database were used for individual-level analyses, while for species-level analyses, we used the body mass available in EltonTraits and Paglia. Missing data on species-level traits were estimated using mode values (the most commonly occurring value) from closely related species within the same genus, except for body mass, which was estimated using the mean value of the genus. The estimated data are indicated in the data file at figshare [42]: https://doi.org/10.6084/m9.figshare.25838281. An overview of the scale of factors for each trait and the number of replications can be found in the Table 1.

**Table 1:** Overview of the scale at which key parameters were measured and their respective replication

Scale of inference for the chance of infection by the *Bartonella* bacteria	Scale at which the factor of interest is applied	Number of replicates at the appropriate scale
Rodent communities	Communities	Eight communities for each diversity index
Rodent individuals	Species	Body length: 17 species
Rodent individuals	Species	Tail length/body length: 17 species
Rodent individuals	Species	Age: 16 adult, 8 young
Rodent individuals	Species	Sex: 14 feminine, 15 masculine
Rodent individuals	Species	Locomotor habitat: 1 scansorial, 3 semi-scansorial, 15 ground, 1 semi-aquatic, 2 arboreal
Rodent individuals	Species	Dietary guild: 8 insectivore, 13 herbivore, 2 omnivore
Rodent individuals	Species	Activity period: 3 diurnal, 11 nocturnal, 9 cathemeral
Rodent species	Species	Body mass: 17 species
Rodent species	Species	Locomotor habitat: 1 scansorial, 2 semi-scansorial, 11 ground, 1 semi-aquatic, 2 arboreal
Rodent species	Species	Dietary guild: 5 insectivore, 11 herbivore, 1 omnivore
Rodent species	Species	Activity period: 2 diurnal, 9 nocturnal, 6 cathemeral
Mammal species	Species	Body mass: 108 species
Mammal species	Species	Dietary guild: 27 insectivore, 55 herbivore, 18 omnivore, 8 carnivore
Mammal species	Species	Activity period: 12 diurnal, 76 nocturnal, 20 cathemeral

Individual hosts are subsamples that contribute to the precision of the estimates at the species level but are not independent replicates. Therefore, we included the species name as a random effect in the statistical models to avoid pseudoreplication. Further details on the data used in the models can be found in the Additional file: Table S1, and the raw data is available on figshare [40]: https://doi.org/10.6084/m9.figshare.25838281

### Host community structure

Diversity indices representing different aspects of community structure and composition were calculated for each rodent community using Rényi’s diversity profiles, Fisher’s alpha [43], Rao’s quadratic entropy [44], FDis [45], FRic, and FEve [46]. The Rényi diversity profile is a technique for ordering diversity [47] that produces curves indicating the richness and evenness of each community. Fisher’s alpha is a scale-independent biodiversity indicator based on the curvature of the species abundance distribution. Rao’s quadratic entropy is a dissimilarity metric used within a functional space that measures the abundance-weighted sum of pairwise functional distances between all species. FDis is another dissimilarity metric that incorporates the abundance-weighted distance of species trait values from the community centroid. FRic measures the volume of the multidimensional space occupied by all species, while FEve assesses the regularity of the trait distribution and relative species abundance. All diversity metrics were calculated using the packages Vegan [48], BiodiversityR [49], and FD [50] available in R version 4.1.0 [51].

### Brazilian mammal host and *Bartonella* lineage network

We compiled a second dataset using existing GenBank data. All metadata for *Bartonella* sequences, including host name, gene, locality, GenBank accession number, and reference, where the host species was identified, were downloaded. This information was obtained using Geneious software from the NCBI database [52], and all *Bartonella* sequences recorded in Brazil were recovered. For sequences with no scientific host name provided, we obtained information on *Bartonella* hosts from the articles in which the sequences were published (available in the Data Sources section). Data on all mammal species that tested negative for *Bartonella* DNA were also gathered from these articles.

In the present study, the term “genotype” refers specifically to the variant forms of the partial sequences of the genes *gltA*, *rpoB*, *ftsZ*, *groEL*, *nuoG*, *ITS*, *pap31*, *16S*, *ribC,* and *htrA* carried by *Bartonella* bacteria. *Bartonella* genotypes were classified into lineages on the basis of the phylogenies available in the peer-reviewed literature from which the sequences were published (see the Data Sources section). Specifically, *Bartonella* genotypes closely related to species that occur in Brazil, such as *Bartonella coopersplainsensis*, *B. bovis*, *B. clarridgeiae*, *B. rochalimae*, *B. quintana*, *B. henselae*, *B. koehlerae*, and *B. vinsonii*, were classified into lineages as indicated by Wagner and Dehio [22]. This classification facilitates the analysis of how *Bartonella* lineages are shared among hosts, as these lineages exhibit patterns of distribution of virulence factors that affect their pathogenicity and host adaptation [22].

### Host phylogenetic distance

At the rodent individual scale, we used phylogenetic distances estimated from the Atlantic Forest non-volant small mammal tree [53]. We incorporated cytochrome B sequences from three rodent species not included in this tree to complete the final phylogenetic distance matrix. Sequence alignment and pairwise distances for each species were calculated using the maximum likelihood method with 500 bootstrap replications, assuming Gamma distribution with invariant sites and a very strong branch swap filter in MEGA^©^ software (www.megasoftware.net). The phylogenetic tree was built using the Kimura 2-parameter method, which is commonly used for estimating genetic distances and phylogenetic relationships [54].

At the species scale, we used the cophenetic.philo function of the ape package [55] to calculate the phylogenetic distances between Brazilian mammal species with PCR-positive or negative *Bartonella* detections. We sampled 10,000 equally plausible mammal phylogenetic trees from the posterior distribution published by Upham et al. [56], covering 108 species. We then created a rooted consensus tree using TreeAnnotator v1.10, summarizing it with a burn-in of the first 1000 trees and a cutoff of 0.7 posterior probability. The most representative tree was rooted at the midpoint of the clusters. One mammal species detected with *Bartonella*, *Bubalus bubalis* (Cetartiodactyla), was absent from the supertree, as were two species that tested negative for *Bartonella* DNA: *Aotus infulatus* (Primates) and *Sphiggurus villosus* (Rodentia).

### Statistical analyses

#### Diversity indices and odds of *Bartonella* infection for each community

To investigate whether the odds of *Bartonella* infection were affected by the functional diversity of the host community, we used generalized linear models (GLM) from the package lme4 [57] with a binomial family and a logit link function. The significance of the model was evaluated by simulating a null model to test the absence of an effect of functional diversity indices on *Bartonella* infection and evaluated using analysis of variance (ANOVA) at the probability level of 0.05.

### Rodent traits as predictors of *Bartonella* infection

To assess whether rodent sex, age, body length, dietary guild, locomotor habitat, and activity period may drive *Bartonella* infection, we used data from all individuals sampled in the ten communities. Note that, because body length and tail length are highly correlated (Pearson correlation coefficient, *r* = 0.7, *P* < 0.05), we evaluated whether the ratio of these two variables would be a better predictor than body length alone. Conversely, the Spearman correlations between age, sex, and body length were statistically not significant. The exotic synanthropic species *Rattus rattus* and *Mus musculus* were excluded from this analysis.

To test whether host traits influenced infection status while accounting for rodent phylogenetic relatedness, candidate phylogenetic generalized linear mixed models (GLMM) were fitted using the brms package with default priors and infection status as a Bernoulli-distributed response. The identity of rodent species and the phylogenetic covariance matrix were included as random effects [58]. Four chains of 10,000 iterations each were run with a burn-in period of 5000 and thinned every 10 steps, resulting in a total of 4000 samples. GLMMs were compared using leave-one-out cross-validation (LOOIC), and goodness of fit was assessed with Bayesian R^2^, which includes the total modeled variance attributed to fixed effects [59, 60]. Fixed effects [means and 95% highest density intervals (HDI)] were estimated from the posterior distributions of each predictor in the best phylogenetic GLMM.

### Network analysis of all PCR-positive hosts for *Bartonella* in Brazil

To highlight species notable for sharing multiple lineages, we created a bipartite network in which the nodes represent mammalian host species from different orders or *Bartonella* lineages, and the edges among the nodes represent associations between host species and *Bartonella* lineages. We calculated the degree centrality (number of edges connected to a node) and the betweenness centrality (number of shortest paths passing through a node) using the igraph package [61]. These measures describe each host’s role in sharing *Bartonella* lineages. For instance, hosts with high degree centrality play a significant role in spreading parasite diversity, while hosts with high betweenness centrality act as bridges between different groups of hosts, particularly if they have greater contact with other hosts due to similarities in their ecological traits.

The GLMs were fitted to examine whether the host trait patterns affected the host centralities in the network. We used different statistical methods to analyze network centrality measures. For degree centrality, we applied a GLM assuming a Poisson distribution of the data. For betweenness centrality, we transformed the data using a logit function and then used a GLM assuming a normal distribution (Gaussian errors).

### Trait and phylogenetic similarities as predictors

The effects of host ecological and evolutionary similarity on interaction patterns were evaluated using multiple regression on distance matrices [62] with the ecodist package [63]. We examined how phylogenetically similar hosts or those with similar traits share *Bartonella* compared with dissimilar ones. Three pairwise matrices were created for rodents sampled from the Atlantic Forest, which were tested for the detection of *Bartonella* DNA: phylogenetic distance, trait profile distance, and a distance matrix for positivity. The same three pairwise matrices were also created for all mammals tested for the detection of *Bartonella* DNA in Brazil. The distance matrix for positivity, derived from the presence or absence of *Bartonella* in each host species, was constructed using Jaccard’s qualitative index [64].

### Data source and R code

A list of data and R code used in the study are provided at figshare [42]: https://doi.org/10.6084/m9.figshare.25838281. A list of all included studies can be found in the Data Sources section.

## Results

### Structures of the rodent community and the chances of infection with *Bartonella*

Six host species with *Bartonella* DNA were detected in eight of the ten analyzed communities, as follows: *Akodon cursor* (87 individuals caught, 75 tested, 15 PCR positive), *Akodon montensis* (21 caught, 3 tested, 2 PCR positive), *Delomys dorsalis* (62 caught, 14 tested, 3 PCR positive), *Euryoryzomys russatus* (9 caught, 7 tested, 6 PCR positive), *Nectomys squamipes* (33 caught, 30 tested, 3 PCR positive), and *Oxymycterus dasytrichus* (20 caught, 14 tested, 3 PCR positive). A summary of the data collected used in our models is provided in the Additional file: Table S1. The Rényi diversity profile (Additional file: Fig. S1) showed that the communities’ richness indices (alpha = 0), Shannon (alpha = 1), and Simpson (alpha = 2) had overlapping confidence intervals, making comparison using classical diversity metrics difficult.

Using data from eight study sites, our findings show that Fisher’s alpha ranged between 1.64 and 2.52, and functional diversity varied among communities as follows: FRic (0.007–0.26), FEve (0.41–0.91), RaoQ (0.05–0.17), and FDis (0.17–0.41). Our models did not indicate significant relationship between functional diversity and the chances of *Bartonella* infection in the rodent communities investigated (Additional file: Table S2).

### Host trait: predictors at the individual level

**Figure 2:**
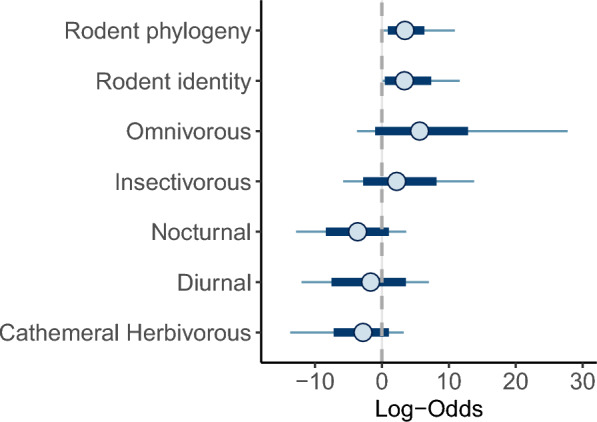
Predictors of *Bartonella* infection in rodent. The graph illustrates the effect of the dietary guild and the activity period on the odds of *Bartonella* infection in Atlantic Forest rodents. The uncertainty of these effects is represented by the width of the credible interval. Posterior means of the odds of *Bartonella* infection are shown, with 80% HDI (thick segments) and 95% HDI (thinner outer lines) from the most parsimonious phylogenetic GLMM. The reference levels for the chances of *Bartonella* infection are cathemeral and herbivorous rodents

The best phylogenetic GLMM was fitted with binary data on the presence or absence of *Bartonella* as dependent variable, with dietary guild and activity period as fixed effects. We excluded the locomotor habitat from the models due to overfitting. The complete ranking of the candidate models is shown in the Additional file: Table S3. We observed a weak effect of the dietary guild and the activity period on the odds of *Bartonella* infection in Atlantic Forest rodents, with this effect characterized by considerable uncertainty, as indicated by the credible interval overlapping zero (Fig. 2). There was no statistical support for the influence of individual-level morphological traits or species-level ecological traits on *Bartonella* infection, after accounting for species identity and phylogeny as random effects (Additional file: Table S3, Fig. 2).

### Influence of traits and phylogenetic distance at the species level

Although we expected that trait and phylogenetic similarities would increase opportunities for contact with susceptible species and thus enhance *Bartonella* infection, no significant effect was detected at the species level (Additional file: Table S4). This finding remained consistent when considering phylogenetic proximity in the *Bartonella* infection patterns among all mammal hosts listed for Brazil (Additional file: Table S4).

### Brazilian host-*Bartonella* lineage network

**Figure 3:**
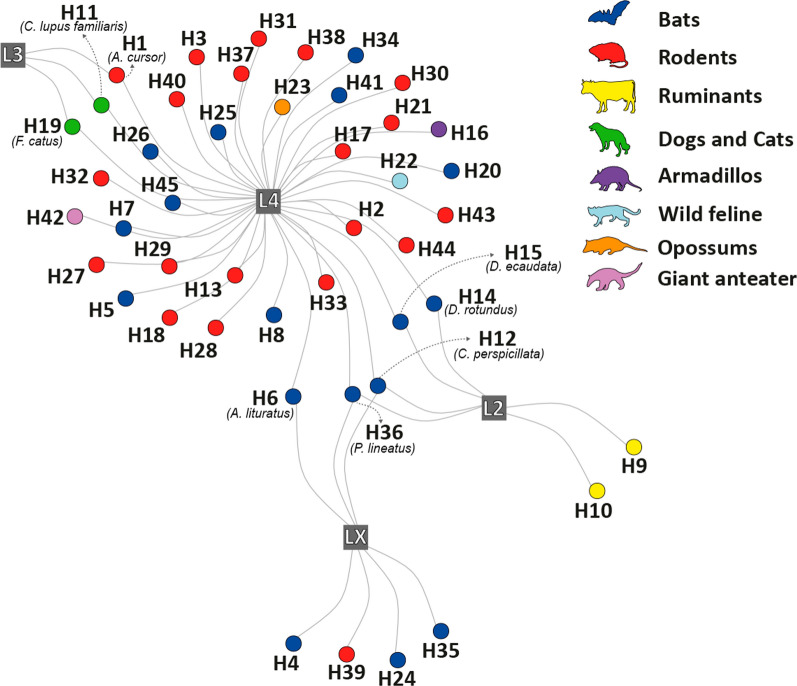
Bipartite network of interactions between mammal species (circles) and *Bartonella* lineages (squares) found in Brazil. One node represents a lineage with no phylogenetic tree classification (LX) identified in seven host species reported in the studies used to build the interaction network. Lineage 4 (L4) is the most promiscuous, detected in 36 of the 45 mammal species known as hosts of *Bartonella* in Brazil. Lineage 2 (L2) has been detected in six species, including two ruminants and four bats. *Canis lupus familiaris* (H11), *Felis catus* (H19), and *Akodon cursor* (H1) share L3 and L4. *Carollia perspicillata* (H12) and *Desmodus rotundus* (H14), *Diphylla ecaudata* (H15), and *Platyrrhinus lineatus* (H36) share L2 and L4. Other host species share L4, as follows: H2. *Akodon montensis*; H3. *Akodon* sp.*;* H4. *Anoura caudifer*; H5. *Artibeus fimbriatus*; H6. *Artibeus lituratus*; H7. *Artibeus obscurus;* H8. *Artibeus planirostris;* H9. *Bos taurus*; H10. *Bubalus bubalis*; H13. *Delomys dorsalis*; H16. *Euphractus sexcinctus*; H17. *Euryoryzomys macconnelli*; H18. *Euryoryzomys russatus*; H20. *Glossophaga soricina*; H21. *Hylaeamys megacephalus*; H22. *Leopardus geoffroyi*; H23. *Marmosops ocellatus*; H24. *Myotis izecksohni*; H25. *Myotis riparius*; H26. *Myotis* sp.; H27. *Neacomys spinosus*; H28. *Necromys lasiurus*; H29. *Nectomys squamipes*; H30. *Oecomys mamorae*; H31. *Oligoryzomys nigripes*; H32. *Oxymycterus dasytrichus*; H33. *Oxymycterus nasutus*; H34. *Phyllostomus discolor*; H35. *Phyllostomus* sp.; H37. *Proechimys gardneri*; H38. *Rattus norvegicus*; H39. *Rattus rattus*; H40. *Rhipidomys macrurus*; H41.* Sturnira lilium*; H42. *Tamandua tetradactyla*; H43. *Thrichomys fosteri*; H44. *Thrichomys laurentius*; H45. *Uroderma bilobatum.* Three sequences were not used in the network, as their lineage classifications were either not found in the primary article or because the host’s scientific name was not reported, namely KY356756 (host not reported), MG878887, and MG878888 (*Glossophaga soricina* as host)

Considering *Bartonella* lineages instead of genotypes allowed us to explore a broader aspect of the association between these proteobacteria and its hosts. In general, *Bartonella* was detected in at least one species of six mammal orders in Brazil, with the bacteria most commonly found in rodent and bat species. The *Bartonella* lineage could not be determined for eight bat species and three rodent species. This network illustrates which *Bartonella* lineages have more widespread host distributions, and the network node properties suggest that the Chiroptera order likely plays a major role in sharing the *Bartonella* lineage with other host orders, due to its higher betweenness centrality. The degree centrality was highest for the common vampire bat (*Desmodus rotundus*; H14) and the hairy-hegged vampire bat (*Diphylla ecaudata*; H15), the cursor grass mouse (*A. cursor*; H1), the great fruit-eating bat (*Artibeus lituratus*; H6), the domestic dog (*Canis lupus familiaris*; H11), and the domestic cat (*Felis catus*; H19), indicating that these host species interacted with two *Bartonella* lineages in the host–parasite network (Fig. 3). Seba’s short-tailed bat (*Carollia perspicillata*; H12) and white-lined broad-nosed bat (*Platyrrhinus lineatus*; H36) also presented the highest degree (3) and the betweenness centrality values (Additional file: Table S5). Although host traits were not associated with network centralities (see Additional file: Tables S6-S7), bats with higher centrality had overlapped activity periods with *A*. *cursor,* and domestic dogs and cats, since these species are nocturnal and cathemeral. *D*. *rotundus* and *D*. *ecaudata* are carnivorous, similar to domestic host species. *C*. *perspicillata*, *P*. *lineatus*, and *A*. *lituratus* are herbivorous, but like *A*. *cursor*, they also include invertebrates in their diet.

## Discussion

In Brazil, eco-epidemiological studies on *Bartonella* infection in rodents have been mainly concentrated in the Atlantic Forest region [36–38]. Here, we anticipated that the functional diversity of rodent communities sampled across the Atlantic Forest would affect the odds of *Bartonella* infection. However, variations in functional diversity measures—namely FRic, FEve, RaoQ, and FDis—did not explain the odds of *Bartonella* infection in these rodent communities. This could be due to the high functional redundancy found in non-volant small mammal communities with more than five species in the Atlantic Forest [53]. With several species exhibiting similar trait values, the impact of functional diversity on transmission dynamics may be mitigated, as multiple species can fulfill similar ecological roles. Another hypothesis is that the density of host populations and their contact rates with vectors play a more critical role in *Bartonella* transmission than community structure itself. This indicates that control measures should prioritize reducing humans-vector contact rather than focusing solely on community composition.

Other components of community diversity, such as species richness and Shannon index, have previously been linked to the prevalence of *Bartonella* infections in various regions of the USA [65]. However, it was unclear whether these indices accounted for the different sampling efforts across the analyzed communities, which is crucial for inter-site biodiversity comparisons. In our study, we included the sampling effort as a covariate in a GLM model and found no association between species richness or the Shannon index and the chances of *Bartonella* infection. Additionally, our findings may be influenced by environmental factors that were not account in our analyses, as well as the presence of vectors. Vectors is a key determinant in the spread of *Bartonella* within host communities [66]. Environmental factors, such as temperature and humidity, also influence the abundance of fleas [67]. Therefore, more comprehensive studies that integrate environmental parameters with vector presence are essential to elucidate the prevalence of *Bartonella* in host communities.

In this study, we anticipated that cathemeral and insectivorous hosts would be associated with increased *Bartonella* infection. However, we found no statistical support for the influence of morphological and ecological traits on *Bartonella* infection in rodents. These expected relationships may be obscured by the complex interactions between host behaviour, vector activity, and habitat use. Host exposure is related to encounter rates between hosts and vectors. Fleas that transmit *Bartonella* are most active during the daytime [68], making cathemeral (partially daytime) and diurnal (fully daytime) hosts more susceptible to infection. Additionally, while oral transmission of *Bartonella* in rodents is less effective in causing bacteremia [69], insectivorous rodent species may have increased exposure to *Bartonella* vectors in their foraging habitats. For instance, ticks are commonly found ectoparasites in environments where insectivorous rodents live, such as forests and grasslands [32, 33]. Therefore, surveillance efforts should also consider the diversity of habitats that rodents inhabit, including natural, altered, and agricultural areas, as these environments can affect both vector and host populations.

Regarding the interaction network between mammal host species and *Bartonella* lineages, lineage 4 (L4) appears to spread among a diverse range of hosts compared with other lineages. This suggests that L4 has a broad potential to infect hosts that are phylogenetically distant and occupy different ecological niches. While *Bartonella* genotypes often cluster by mammal taxonomic orders [70], this can obscure high host specificity when investigating at the lineage level, where genotypes closely related to a particular lineage are grouped together. The specificity of *Bartonella* genotypes suggests greater biological restrictions at this level, but the classification of lineages, as described by Wagner and Dehio [22], reveals important patterns in the distribution of pathogenicity-related virulence factors among lineages. It is worth noting that the unknown *Bartonella* lineages (represented in the network as LX) may not constitute a single lineage and may not fit into known lineages. In Brazil, most *Bartonella* infection records are at the genotype level, with limited strain isolation or species delimitation. This limits our ability to classify *Bartonella* species and trace ecological drivers in mammal hosts. Additionally, some hosts and vectors can harbor multiple genotypes, and strains may recombine, affecting genetic similarity among genotypes [71].

Our network analyses aimed to characterize the centrality of mammal species in the sharing of *Bartonella* lineages recorded in Brazil. Some bat species share *Bartonella* lineages with cattle and other bats (see, e.g., [72, 73]), while synanthropic rodent species and domestic animals share two *Bartonella* lineages (L3 and L4). The results highlight key rodent and bat species that should be targeted in eco-epidemiological studies due to their propensity to circulate among domestic animals and share their habitats.

Our findings also suggest that bats may possess traits not examined in this study that make them more prone to various *Bartonella* lineages. It is possible that other ecological traits, such as occupying a greater diversity of roosting habitats, increase the risk of *Bartonella* exposure between bat species [74]. Although *Bartonella* lineages encompass a diverse array of species and genotypes, these bacteria are functionally convergent, possessing factors related to pathogenicity and the capacity to infect host cells [75]. For instance, specific sets of Bep repertoires have adapted to three different *Bartonella* lineages, demonstrating remarkable host adaptation [22]. Consequently, factors such as body mass and ecological traits of the host may have a weaker effect on *Bartonella* infections compared with molecular–host cell interactions.

## Conclusions

Understanding the transmission pathways that drive *Bartonella* infection has significant methodological and zoonotic disease management implications. In Brazil, a continental-sized country, the number of host communities investigated for these bacteria is still very limited. Functional diversity indices require uniformity in comparative data. Although there was no sufficient statistical support to establish trait-based indices, analyses in different biomes should assess the effect of these diversity indices on pathogen infection. The absence of accurate specimen identification and inventory data, such as sampling effort and information on all hosts tested for the detection of *Bartonella,* including those negative for the pathogen, complicates meta-analyses at the scale of host communities. Consequently, study programs focusing on the role of host trait diversity in modulating pathogen infection could provide a semi-quantitative tool for indicator-based surveillance, species management, and control strategies. Regarding all mammal species, no effect of host traits and phylogeny was observed on the sharing of *Bartonella* lineage. An open question is whether molecular–host cell interactions play a more significant role in infection dynamics than host body mass and ecological traits. Identifying key species in *Bartonella* transmission will help guide policies in the human and animal health sectors by informing cross-sectional surveillance efforts. This work highlights wild host species that share *Bartonella* lineages with livestock, synanthropic rodent species, and domestic animals, underscoring the complexity of the maintenance cycle of these proteobacteria within the One Health framework.

## Data sources

- André, M. R., Denardi, N. C. B., de Sousa, K. C. M., Gonçalves, L. R., Henrique, P. C., Ontivero, C. R. G. R., Ontivero, C. R. G. R., Gonzalez, I. H. L., Nery, C. V. C., Chagas, C. R. F., Monticelli, C., Santis, A. C. G. A., & Machado, R. Z. (2014). Arthropod-borne pathogens circulating in free-roaming domestic cats in a zoo environment in Brazil. *Ticks and Tick-borne Diseases*, *5*(5), 545-551. https://doi.org/10.1016/j.ttbdis.2014.03.011
- André, M. R., Dumler, J. S., Herrera, H. M., Gonçalves, L. R., de Sousa, K. C., Scorpio, D. G., Santis, A. C. G. A., Domingos, I. H., De Macedo, G. C., & Machado, R. Z. (2016). Assessment of a quantitative 5'nuclease real-time polymerase chain reaction using the nicotinamide adenine dinucleotide dehydrogenase gamma subunit (nuoG) for *Bartonella* species in domiciled and stray cats in Brazil. *Journal of Feline Medicine and Surgery*, *18*(10), 783-790. https://doi.org/10.1177/1098612X15593787
- André, M. R., Canola, R. A. M., Braz, J. B., Perossi, I. F. S., Calchi, A. C., Ikeda, P., Machado, R. Z., Vasconcelos, R. O., & Camacho, A. A. (2019). Aortic valve endocarditis due to *Bartonella clarridgeiae* in a dog in Brazil. *Revista Brasileira de Parasitologia Veterinária*, *28*, 661-670. https://doi.org/10.1590/S1984-29612019078
- André, M. R., Gutiérrez, R., Ikeda, P., Amaral, R. B., Sousa, K. C. M., Nachum‐Biala, Y., Lima, L., Teixeira, M. M. G., Machado, R. Z., & Harrus, S. (2019). Genetic diversity of *Bartonella* spp. in vampire bats from Brazil. *Transboundary and Emerging Diseases*, *66*(6), 2329-2341. https://doi.org/10.1111/tbed.13290
- Bonato, L., Figueiredo, M. A. P., Gonçalves, L. R., Machado, R. Z., & André, M. R. (2015). Occurrence and molecular characterization of *Bartonella* spp. and hemoplasmas in neotropical primates from Brazilian Amazon. *Comparative Immunology, Microbiology and Infectious Diseases*, *42*, 15-20. https://doi.org/10.1016/j.cimid.2015.09.001
- Braga, I. A., Dias, I. S. D. O., Chitarra, C. S., Amude, A. M., & Aguiar, D. M. (2015). Molecular detection of *Bartonella clarridgeiae* in domestic cats from Midwest Brazil. *Brazilian Journal of Infectious Diseases*, *19*, 451-452. https://doi.org/10.1016/j.bjid.2015.05.002
- Calchi, A. C., Vultão, J. G., Alves, M. H., Yogui, D. R., Desbiez, A. L. J., do Amaral, R. B., de Santi, M., Teixeira, M. M. G., Werther, K., Machado, R. Z., & André, M. R. (2020). Multi‐locus sequencing reveals a novel *Bartonella* in mammals from the Superorder Xenarthra. *Transboundary and Emerging Diseases*, tbed.13545. https://doi.org/10.1111/tbed.13545
- de Paiva Diniz, P. P. V., Maggi, R. G., Schwartz, D. S., Cadenas, M. B., Bradley, J. M., Hegarty, B., & Breitschwerdt, E. B. (2007). Canine bartonellosis: serological and molecular prevalence in Brazil and evidence of co-infection with *Bartonella henselae* and *Bartonella vinsonii* subsp. *berkhoffii*. *Veterinary Research*, *38*(5), 697-710. http://doi.org/10.1051/vetres:2007023
- de Sousa, K. C. M., do Amaral, R. B., Herrera, H. M., Santos, F. M., Macedo, G. C., de Andrade Pinto, P. C. E., Barros-Battesti, D. M., Machado, R. Z., & André, M. R. (2018). Genetic diversity of *Bartonella* spp. in wild mammals and ectoparasites in Brazilian Pantanal. *Microbial Ecology*, *76*(2), 544-554. https://doi.org/10.1007/s00248-017-1138-0
- Ferreira, M. S., Guterres, A., Rozental, T., Novaes, R. L. M., Vilar, E. M., Oliveira, R. C. D., Fernandes, J., Forneas, D., Alvino Junior, A., Brandão, M. L., Cordeiro, J. L. P., Alvarez, M. R. D. V., Althoff, S. L., Moratelli, R., Cordeiro-Estrela, P., da Silva, R. C. & Lemos, E. R. S. D. (2018). *Coxiella* and *Bartonella* spp. in bats (Chiroptera) captured in the Brazilian Atlantic Forest biome. *BMC veterinary research*, *14*(1), 1-10. https://doi.org/10.1186/s12917-018-1603-0
- Gonçalves-Oliveira, J., Rozental, T., Guterres, A., Teixeira, B. R., Andrade-Silva, B. E., Costa-Neto, S. F. da, Furtado, M. C., Moratelli, R., D’Andrea, P. S., & Lemos, E. R. S. (2020). Investigation of *Bartonella* spp. in brazilian mammals with emphasis on rodents and bats from the Atlantic Forest. *International Journal for Parasitology: Parasites and Wildlife*, *13*, 80-89. https://doi.org/10.1016/j.ijppaw.2020.07.004
- Gonçalves, L. R., Favacho, A. R. de M., Roque, A. L. R., Mendes, N. S., Fidelis Junior, O. L., Benevenute, J. L., Herrera, H. M., D’Andrea, P. S., de Lemos, E. R. S., Machado, R. Z., & André, M. R. (2016). Association of *Bartonella* species with wild and synanthropic rodents in different Brazilian biomes. *Applied and Environmental Microbiology*, *82*(24), 7154-7164. https://doi.org/10.1128/AEM.02447-16
- Gonçalves, L. R., Harrus, S., Gutiérrez, R., Herrera, H. M., Souza Ramos, I. A., Porfírio, G. E. de O., Nachum‐Biala, Y., Sousa, K. C. M., Silva, T. M. V., Campos, J. B. V., Lemos, W., Moraes Barros-Battesti, D., Machado, R. Z., & André, M. R. (2020). Molecular detection and genetic diversity of *Bartonella* species in large ruminants and associated ectoparasites from the Brazilian Cerrado. *Transboundary and Emerging Diseases*, tbed.13517. https://doi.org/10.1111/tbed.13517
- Gonçalves, L. R., Harrus, S., Herrera, H. M., Gutierrez, R., Pedrassani, D., Nantes, W. A. G., Santos, F. M., Porfírio, G. E. O., Barreto, W. T. G., Macedo, G. C., Assis, W. O., Campos, J. B. V., Silva, T. M. V., Biolchi, J., Sousa, K. C. M., Nachum-Biala, Y. N., Barros-Battesti, D. M., Machado, R. Z., & André, M. R. (2020). Low occurrence of *Bartonella* in synanthropic mammals and associated ectoparasites in peri-urban areas from Central-Western and Southern Brazil. *Acta Tropica*, *207*, 105513. https://doi.org/10.1016/j.actatropica.2020.105513
- Hayman, D. T., McDonald, K. D., & Kosoy, M. Y. (2013). Evolutionary history of rat-borne *Bartonella*: the importance of commensal rats in the dissemination of bacterial infections globally. *Ecology and Eolution*, *3*(10), 3195-3203. https://doi.org/10.1002/ece3.702
- Ikeda, P., Seki, M. C., Carrasco, A. O. T., Rudiak, L. V., Miranda, J. M. D., Gonçalves, S. M. M., Hoppe, E. G. L., Albuquerque, A. C. A., Teixeira, M. M. G., Passos, C. E., Werther, K., Machado, R. Z., & André, M. R. (2017). Evidence and molecular characterization of *Bartonella* spp. and hemoplasmas in neotropical bats in Brazil. *Epidemiology and Infection*, *145*(10), 2038-2052. https://doi.org/10.1017/S0950268817000966
- Ikeda, P., Marinho Torres, J., Perles, L., Lourenço, E. C., Herrera, H. M., de Oliveira, C. E., Zacarias Machado, R., & André, M. R. (2020). Intra- and inter-host assessment of *Bartonella* diversity with focus on non-hematophagous bats and associated ectoparasites from Brazil. *Microorganisms*, *8*(11), 1822. https://doi.org/10.3390/microorganisms8111822
- Miceli, N. G., Gavioli, F. A., Gonçalves, L. R., André, M. R., Sousa, V. R. F., Sousa, K. C. M. D., & Machado, R. Z. (2013). Molecular detection of feline arthropod-borne pathogens in cats in Cuiabá, state of Mato Grosso, central-western region of Brazil. *Revista Brasileira de Parasitologia Veterinária*, *22*, 385-390. https://doi.org/10.1590/S1984-29612013000300011
- Pedrassani, D., Biolchi, J., Gonçalves, L. R., Mendes, N. S., Zanatto, D. C. D. S., Calchi, A. C., Machado, R. Z., & André, M. R. (2019). Molecular detection of vector-borne agents in cats in Southern Brazil. *Revista Brasileira de Parasitologia Veterinária*, *28*, 632-643. https://doi.org/10.1590/S1984-29612019077
- Rozental, T., Ferreira, M. S., Guterres, A., Mares-Guia, M. A., Teixeira, B. R., Gonçalves, J., Bonvicino, C. R., D’Andrea, P. S., & de Lemos, E. R. S. (2017). Zoonotic pathogens in Atlantic Forest wild rodents in Brazil: *Bartonella* and *Coxiella* infections. *Acta Tropica*, *168*, 64-73. https://doi.org/10.1016/j.actatropica.2017.01.003
- Silva, B. T. G. D., Souza, A. M. D., Campos, S. D. E., Lemos, E. R. S. D., Favacho, A. R. D. M., & Almosny, N. R. P. (2018). Presence of *Bartonella* spp. in domestic cats from a state park in Rio de Janeiro, Brazil. *Revista do Instituto de Medicina Tropical de São Paulo*, *60*. https://doi.org/10.1590/S1678-9946201860014
- Souza, A. M., Almosny, N. R. P., Favacho, A. R. M., Almeida, D. N. P., Ferreira, R. F., Ferreira, E. O., Moreira, N; S., & Lemos, E. R. S. (2017). *Bartonella* spp. and hematological changes in privately owned domestic cats from Rio de Janeiro, Brazil. *The Journal of Infection in Developing Countries*, *11*(08), 591-596. https://doi.org/10.3855/jidc.8152
- Souza, U. A., Webster, A., Dall’Agnol, B., Morel, A. P., Peters, F. B., Favarini, M. O., Mazim, F. D., Soares, J. B. G., Tirelli, F. P., Tortato, M. A., de Lemos, E. R. S., Trigo, T. C., Soares, J. F., & Reck, J. (2021). Molecular and serological survey of the cat-scratch disease agent (*Bartonella henselae*) in free-ranging *Leopardus geoffroyi* and *Leopardus wiedii* (Carnivora: Felidae) from Pampa biome, Brazil. *Microbial Ecology*, *81*(2), 483-492. https://doi.org/10.1007/s00248-020-01601-x
- Staggemeier, R., Venker, C. A., Klein, D. H., Petry, M., Spilki, F. R., & Cantarelli, V. V. (2010). Prevalence of *Bartonella henselae* and *Bartonella clarridgeiae* in cats in the south of Brazil: a molecular study. *Memórias do Instituto Oswaldo Cruz*, *105*, 873-878. https://doi.org/10.1590/S0074-02762010000700006

## Supplementary Information

Additional file 1: Fig. S1 Rényi’s diversity profiles of the rodent communities sampled in ten municipalities in Rio de Janeiro state, Brazil. Table S1 Summary of the number of communities, individuals and species studied at the three different levels of the analyses. Table S2 Functional diversity indices models using the odds ratio of *Bartonella* infection (logit-transformed) as the response variable between rodent communities in the state of Rio de Janeiro, Brazil. Table S3 Phylogenetic generalized linear mixed models predicting the status of *Bartonella* infection between rodent individuals of the Atlantic Forest (*n* = 192 after removing missing values). Table S4 Multiple regression coefficients for species interaction distance matrices, considering the presence and absence of *Bartonella* per host species and their phylogenetic and trait profile distances. Table S5 Properties of network node: values of degree and betweenness centralities. Table S6 Full ranking of candidate generalized linear models predicting degree centrality. Table S7 Full ranking of candidate generalized linear models predicting betweenness centrality.

## Data Availability

The datasets supporting the conclusions of this article are available in the figshare repository [42]: https://doi.org/10.6084/m9.figshare.25838281. Additionally, a list of data sources used in the study are provided in the Data sources section.
