# Supplementary material for: From host individual traits to community structure and composition: Bartonella infection insights
Source: Parasit Vectors. 2024 Oct 28;17:440. doi: 10.1186/s13071-024-06523-y (PMC11514747; doi:10.1186/s13071-024-06523-y)
Supplement: Supplementary file 1 — Additional file 1: Fig. S1 Rényi’s diversity profiles of the rodent communities sampled in ten municipalities in Rio de Janeiro state, Brazil. Table S1 Summary of the number of communities, individuals and species studied at the three different levels of the analyses. Table S2 Functional diversity indices models using the odds ratio of Bartonella infection (logit-transformed) as the response variable between rodent communities in the state of Rio de Janeiro, Brazil. Table S3 Phylogenetic generalized linear mixed models predicting the status of Bartonella infection between rodent individuals of the Atlantic Forest (n = 192 after removing missing values). Table S4 Multiple regression coefficients for species interaction distance matrices, considering the presence and absence of Bartonella per host species and their phylogenetic and trait profile distances. Table S5 Properties of network node: values of degree and betweenness centralities. Table S6 Full ranking of candidate generalized linear models predicting degree centrality. Table S7 Full ranking of candidate generalized linear models predicting betweenness centrality. [file 13071_2024_6523_MOESM1_ESM.docx]

**TITLE:** From host individual traits to community structure and composition: *Bartonella* infection insights

Gabriella Lima Tabet Cruz, Jonathan Gonçalves-Oliveira, Elba Regina Sampaio de Lemos, Paulo Sergio D’Andrea, Cecilia Siliansky de Andreazzi

**SUPPORTING INFORMATION**

**Fig. S1** Rényi’s diversity profiles of the rodent communities sampled in 10 municipalities in Rio de Janeiro state, Brazil. This graph shows considerable overlap in the confidence intervals, but some communities are ordered by average diversity. The profile value for alpha = 1 represents the Shannon Diversity Index, and the value for alpha = 2 represents the logarithm of the Simpson Diversity Index (1/D). At scale Inf, communities are ordered by the proportion of dominant species, with more equitable communities showing lower values. Similarly to species accumulation curves, the result reflects diversity based on the same number of sample efforts for each level of a categorical variable. This is achieved by calculating a diversity accumulation surface for each community and selecting profiles for the number of samples from the community with the fewest sample efforts (see Kindt & Coe, 2005 for details).

**Table S1** Summary of the number of communities, individuals and species studied at the three different levels of the analyses. More details of the raw data can be found at figshare: https://[doi.org/](https://figshare.com/s/9da764dc83708d110b0c)10.6084/m9.figshare.25838281.

**Table S2** Functional diversity indices models using the odds ratio of *Bartonella* infection (logit-transformed) as the response variable between rodent communities in the state of Rio de Janeiro, Brazil. None of these models was more plausible than the null model to predict *Bartonella* lineage 4 infection.

**Table S3** Phylogenetic generalized linear mixed models predicting the status of *Bartonella* infection between rodent individuals of the Atlantic Forest (n = 192 after removing missing values). Traits are predictor variables, and the presence or absence of *Bartonella* is the response variable (logit-transformed). ‘A’ represents the phylogenetic covariance matrix, and ‘sp’ denotes the identity of the rodent, both included as random effects. Models are ranked by ELPD (expected log pointwise predictive density) with their ELPD SE (standard error), LOOIC (leave-one-out information criterion) with their LOOIC SE (standard error), and Bayesian R² estimates (marginal and conditional on random effects).

**Table S4** Multiple regression coefficients for species interaction distance matrices, considering the presence and absence of *Bartonella* per host species and their phylogenetic and trait profile distances. This analysis includes sampled rodents from the analysed communities and all Brazilian mammal hosts.

**Table S5** Properties of network node: values of degree and betweenness centralities.

**Table S6** Full ranking of candidate Generalized Linear Models predicting degree centrality. These models, built using data from the Brazilian mammal-*Bartonella* lineage network, are ranked by ΔAICc, with degrees of freedom (df) and Akaike weights (wi). Host traits are used as predictive variables, and the presence or absence of *Bartonella* serves as the response variable (logit transformed).

**Table S7** Full ranking of candidate generalized linear models predicting betweenness centrality. These models, built using data from the Brazilian mammal-*Bartonella* lineage network, are ranked by ΔAICc, with degrees of freedom (df) and Akaike weights (wi). Host traits are used as predictive variables, and the presence or absence of *Bartonella* serves as the response variable (logit transformed).


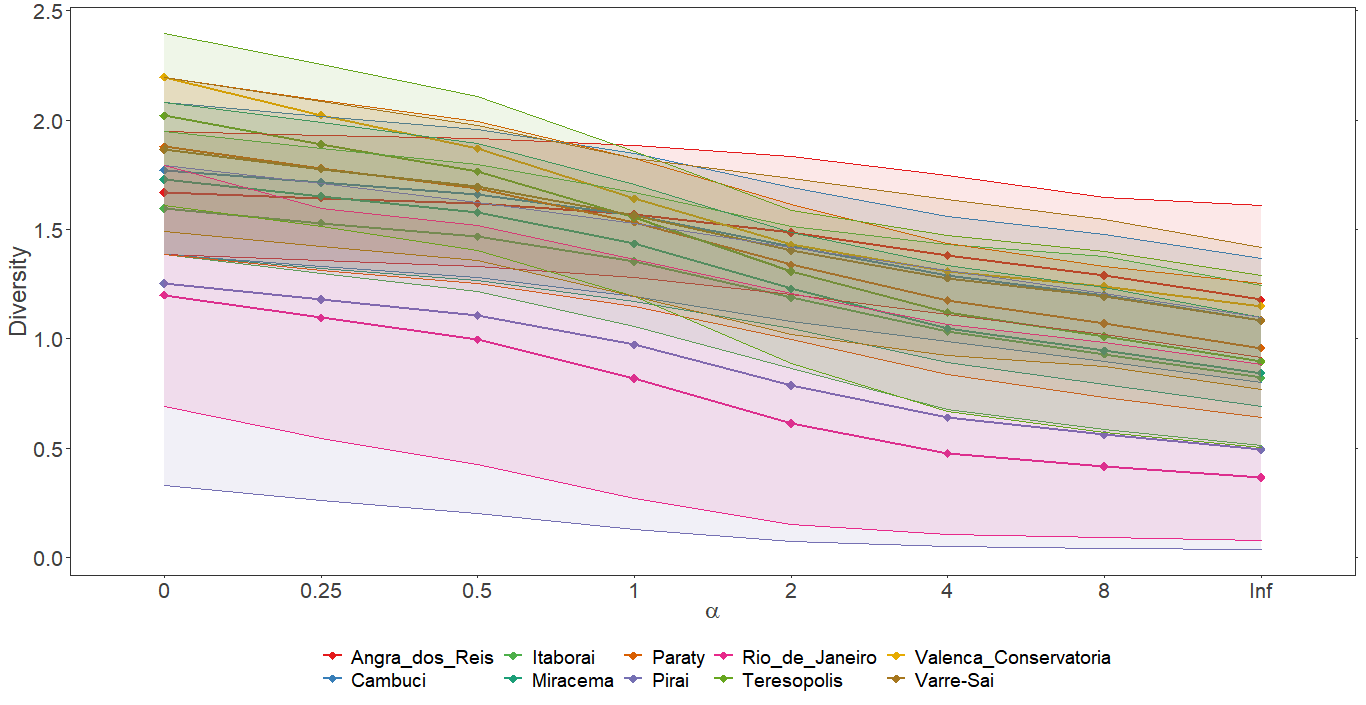


**Fig. S1** Rényi’s diversity profiles of the rodent communities sampled in 10 municipalities in Rio de Janeiro state, Brazil. This graph shows considerable overlap in the confidence intervals, but some communities are ordered by average diversity. The profile value for alpha = 1 represents the Shannon Diversity Index, and the value for alpha = 2 represents the logarithm of the Simpson Diversity Index (1/D). At scale Inf, communities are ordered by the proportion of dominant species, with more equitable communities showing lower values. Similarly to species accumulation curves, the result reflects diversity based on the same number of sample efforts for each level of a categorical variable. This is achieved by calculating a diversity accumulation surface for each community and selecting profiles for the number of samples from the community with the fewest sample efforts (see Kindt & Coe, 2005 for details).

**Table S1** Summary of the number of communities, individuals and species studied at the three different levels of the analyses. More details of the raw data can be found at figshare: https://[doi.org/](https://figshare.com/s/9da764dc83708d110b0c)10.6084/m9.figshare.25838281.

| **Scale of inference** | ****‍Captured**** | ****Tested for the detection of *Bartonella* DNA**** | ****Positive detection of *Bartonella* DNA**** | ****Excluded from the models**** |
| --- | --- | --- | --- | --- |
| **‍‍At the community level across the Brazilian Atlantic Forest: rodents** | **‍398 individuals belonging to five families, 15 genera, and 25** **species in 10 studied municipalities.** | **220 individuals (55.27% of the total) from 18 species from 10 communities.** | **32 individuals from six species in eight communities.** | **Two study sites, Itaboraí and Rio de Janeiro, were excluded from the analysis of the rodent community because the FEve cannot be calculated for communities with three or fewer species. Therefore, eight communities were analysed.** |
| **‍‍‍At the individual and species levels in the Brazilian Atlantic Forest: rodents** | **The same as above.** | **‍201 individuals from 17 species, excluding *Mus musculus.*** | **32 individuals from six species.** | **Nine individuals missing information on individual traits; therefore, 192 individuals from 17 species were included in the individual and species-level models.** |
| **‍‍At the species level across Brazil: mammals from various orders** | **‍Not included in the meta-analysis.** | **111 species.** | **45 species.** | **Three species were absent from the supertree phylogeny and, therefore, only 108 species were included in the statistical analyses.** |

**Table S2** Functional diversity indices models using the odds ratio of *Bartonella* infection (logit-transformed) as the response variable between rodent communities in the state of Rio de Janeiro, Brazil. None of these models was more plausible than the null model to predict *Bartonella* lineage 4 infection.

| **Odds ratio ~ index** | **AICc** | **ΔAICc** | **k** |
| --- | --- | --- | --- |
| glm null | 35.5 | 0.0 | 1 |
| RaoQ | 38.7 | 3.1 | 2 |
| Richness:sample effort | 38.8 | 3.3 | 2 |
| Shannon:sample effort | 39.0 | 3.4 | 2 |
| alpha-Fisher | 39.1 | 3.6 | 2 |
| FDis | 39.1 | 3.6 | 2 |
| FEve | 39.2 | 3.7 | 2 |
| Fric | 39.2 | 3.7 | 2 |

**Table S3** Phylogenetic generalized linear mixed models predicting the status of *Bartonella* infection between rodent individuals of the Atlantic Forest (n = 192 after removing missing values). Traits are predictor variables, and the presence or absence of *Bartonella* is the response variable (logit-transformed). ‘A’ represents the phylogenetic covariance matrix, and ‘sp’ denotes the identity of the rodent, both included as random effects. Models are ranked by ELPD (expected log pointwise predictive density) with their ELPD SE (standard error), LOOIC (leave-one-out information criterion) with their LOOIC SE (standard error), and Bayesian R² estimates (marginal and conditional on random effects).

| **Model Structure** | **ELPD** | **ELPD SE** | **LOOIC** | **LOOIC SE** | **R²m** | **R²c** |
| --- | --- | --- | --- | --- | --- | --- |
| ~activity + diet + (1\|gr(phylo, cov = A)) + (1\|sp) | 0.0000 | 0.0000 | 151.8641 | 16.9457 | 0.1366 | 0.4645 |
| ~1 + (1\|gr(phylo, cov = A)) + (1\|sp) | -0.7282 | 1.3489 | 153.3206 | 16.2664 | 0 | 0.461 |
| ~body length + activity + diet + (1\|gr(phylo, cov = A)) + (1\|sp) | -1.0013 | 0.9611 | 153.8668 | 17.3968 | 0.1762 | 0.467 |
| ~ age + sex + body length + (1\|gr(phylo, cov = A)) + (1\|sp) | -2.5547 | 2.3033 | 156.9735 | 17.3582 | 0.0757 | 0.473 |
| ~ age + sex + tail/body length + (1\|gr(phylo, cov = A)) + (1\|sp) | -3.1208 | 2.2647 | 158.1057 | 17.3984 | 0.0363 | 0.483 |

**Table S4** Multiple regression coefficients for species interaction distance matrices, considering the presence and absence of *Bartonella* per host species and their phylogenetic and trait profile distances. This analysis includes sampled rodents from the analysed communities and all Brazilian mammal hosts.

| **Parameters** | **F** | **R²** | **P-value** |
| --- | --- | --- | --- |
| Phylogenetic distance (Atlantic Forest rodents) | 0.3533582 | 0.002630066 | 0.500 |
| Trait profile distance (Atlantic Forest rodents) | 1.17835 | 0.008717003 | 0.257 |
| Phylo Dist + Trait Dist (Atlantic Forest rodents) | 0.5926111 | 0.008832733 | 0.449 |
| Phylogenetic distance (all Brazilian mammals) | 0.271947 | 0.00004708 | 0.829 |
| Trait profile distance (all Brazilian mammals) | 2.756032 | 0.0004920912 | 0.178 |
| Phylo Dist + Trait Dist (all Brazilian mammals) | 1.487839 | 0.00051500 | 0.574 |

**Table S5** Properties of network node: values of degree and betweenness centralities.

| **Id_net** | **Degree** | **Betweenness** | **Host name** |
| --- | --- | --- | --- |
| H12 | 3 | 0.0882 | *Carollia perspicillata* |
| H36 | 3 | 0.0882 | *Platyrrhinus lineatus* |
| H6 | 2 | 0.0549 | *Artibeus lituratus* |
| H1 | 2 | 0.0132 | *Akodon cursor* |
| H11 | 2 | 0.0138 | *Canis lupus familiaris* |
| H14 | 2 | 0.0243 | *Desmodus rotundus* |
| H15 | 2 | 0.0243 | *Diphylla ecaudata* |
| H19 | 2 | 0.0132 | *Felis catus* |
| H31 | 1 | 0 | *Oligoryzomys nigripes* |
| H34 | 1 | 0 | *Phyllostomus discolor* |
| H35 | 1 | 0 | *Phyllostomus* sp. |
| H42 | 1 | 0 | *Tamandua tetradactyla* |
| H20 | 1 | 0 | *Glossophaga soricina* |
| H23 | 1 | 0 | *Marmosops ocellatus* |
| H4 | 1 | 0 | *Anoura caudifer* |
| H40 | 1 | 0 | *Rhipidomys macrurus* |
| H41 | 1 | 0 | *Sturnira lilium* |
| H45 | 1 | 0 | *Uroderma bilobatum* |
| H5 | 1 | 0 | *Artibeus fimbriatus* |
| H7 | 1 | 0 | *Artibeus obscurus* |
| H8 | 1 | 0 | *Artibeus planirostris* |
| H24 | 1 | 0 | *Myotis izecksohni* |
| H25 | 1 | 0 | *Myotis* *riparius* |
| H26 | 1 | 0 | *Myotis* *sp.* |
| H10 | 1 | 0 | *Bubalus bubalis* |
| H13 | 1 | 0 | *Delomys dorsalis* |
| H16 | 1 | 0 | *Euphractus sexcinctus* |
| H17 | 1 | 0 | *Euryoryzomys macconnelli* |
| H18 | 1 | 0 | *Euryoryzomys russatus* |
| H2 | 1 | 0 | *Akodon montensis* |
| H21 | 1 | 0 | *Hylaeamys megacephalus* |

**Table S5** (Continued) Properties of network node: values of degree and betweenness centralities.

| **Id_net** | **Degree** | **Betweenness** | **Host name** |
| --- | --- | --- | --- |
| H22 | 1 | 0 | *Leopardus geoffroyi* |
| H27 | 1 | 0 | *Neacomys spinosus* |
| H28 | 1 | 0 | *Necromys lasiurus* |
| H30 | 1 | 0 | *Oecomys mamorae* |
| H3 | 1 | 0 | *Akodon* sp. |
| H32 | 1 | 0 | *Oxymycterus dasytrichus* |
| H33 | 1 | 0 | *Oxymycterus nasutus* |
| H37 | 1 | 0 | *Proechimys gardneri* |
| H38 | 1 | 0 | *Rattus norvegicus* |
| H39 | 1 | 0 | *Rattus rattus* |
| H43 | 1 | 0 | *Thrichomys fosteri* |
| H44 | 1 | 0 | *Thrichomys laurentius* |
| H9 | 1 | 0 | *Bos taurus* |
| H29 | 1 | 0 | *Nectomys squamipes* |

**Table S6** Full ranking of candidate Generalized Linear Models predicting degree centrality. These models, built using data from the Brazilian mammal-*Bartonella* lineage network, are ranked by ΔAICc, with degrees of freedom (df) and Akaike weights (wi). Host traits are used as predictive variables, and the presence or absence of *Bartonella* serves as the response variable (logit transformed).

| **Model structure** | **ΔAICc** | **df** | **wi** |
| --- | --- | --- | --- |
| ~ 1 | 0.0 | 1 | 0.5292 |
| ~ log body mass | 1.9 | 2 | 0.2014 |
| ~ activity | 3.8 | 3 | 0.0791 |
| ~ dietary guild | 4.5 | 4 | 0.0567 |
| ~ locomotor habitat | 5.0 | 6 | 0.0430 |
| ~ log body mass + activity | 5.8 | 4 | 0.0293 |
| ~ log body mass + dietary guild | 6.4 | 5 | 0.0216 |
| ~ log body mass + locomotor habitat | 7.0 | 7 | 0.0162 |
| ~ dietary guild + activity | 8.4 | 6 | 0.0079 |
| ~ activity + locomotor habitat | 8.7 | 8 | 0.0068 |
| ~ dietary guild + locomotor habitat | 9.7 | 9 | 0.0041 |
| ~ log body mass + dietary guild + activity | 10.4 | 7 | 0.0030 |
| ~ log body mass + dietary guild + locomotor habitat | 11.7 | 10 | 0.0015 |
| ~ log body mass + dietary guild + locomotor habitat + activity | 15.3 | 12 | <0.0001 |

**Table S7** Full ranking of candidate generalized linear models predicting betweenness centrality. These models, built using data from the Brazilian mammal-*Bartonella* lineage network, are ranked by ΔAICc, with degrees of freedom (df) and Akaike weights (wi). Host traits are used as predictive variables, and the presence or absence of *Bartonella* serves as the response variable (logit transformed).

| **Model structure** | **ΔAICc** | **df** | **wi** |
| --- | --- | --- | --- |
| ~ 1 | 0.0 | 2 | 0.4825 |
| ~ log body mass | 1.8 | 3 | 0.1991 |
| ~ activity | 2.8 | 4 | 0.1201 |
| ~ dietary guild | 3.4 | 5 | 0.0894 |
| ~ log body mass + activity | 4.8 | 5 | 0.0443 |
| ~ log body mass + dietary guild | 4.9 | 6 | 0.0411 |
| ~ dietary guild + activity | 6.7 | 7 | 0.0166 |
| ~ log body mass + dietary guild + activity | 8.6 | 8 | 0.0066 |
| ~ locomotor habitat | 15.7 | 7 | <0.001 |
| ~ log body mass + locomotor habitat | 17.7 | 8 | <0.001 |
| ~ dietary guild + locomotor habitat | 18.9 | 10 | <0.001 |
| ~ activity + locomotor habitat | 19.5 | 9 | <0.001 |
| ~ log body mass + dietary guild + locomotor habitat | 20.8 | 11 | <0.001 |
| ~ log body mass + dietary guild + locomotor habitat + activity | 23.8 | 13 | <0.001 |
